# A Design of 10-Bit Asynchronous SAR ADC with an On-Chip Bandgap Reference Voltage Generator

**DOI:** 10.3390/s22145393

**Published:** 2022-07-19

**Authors:** Deeksha Verma, Khuram Shehzad, Sung Jin Kim, Young Gun Pu, Sang-Sun Yoo, Keum Cheol Hwang, Youngoo Yang, Kang-Yoon Lee

**Affiliations:** 1 Department of Electrical and Computer Engineering, Sungkyunkwan University, Suwon 16419, Korea; deeksha27@skku.edu (D.V.); khuram1698@skku.edu (K.S.); sun107ksj@skku.edu (S.J.K.); hara1015@skku.edu (Y.G.P.); rapter@kaist.ac.kr (S.-S.Y.); khwang@skku.edu (K.C.H.); yang09@skku.edu (Y.Y.); 2 SKAIChips Co., Ltd., Suwon 16419, Korea

**Keywords:** asynchronous SAR logic and comparator clock generator, bandgap reference voltage generator, two-stage dynamic comparator, low power consumption

## Abstract

A proposed prototype of a 10-bit 1 MS/s single-ended asynchronous Successive Approximation Register (SAR) Analog-to-Digital Converter (ADC) with an on-chip bandgap reference voltage generator is fabricated with 130 nm technology. To optimize the power consumption, static, and dynamic performance, several techniques have been proposed. A dual-path bootstrap switch was proposed to increase the linearity sampling. The Voltage Common Mode (VCM)-based Capacitive Digital-to-Analog Converter (CDAC) switching technique was implemented for the CDAC part to alleviate the switching energy problem of the capacitive DAC. The proposed architecture of the two-stage dynamic latch comparator provides high speed and low power consumption. Moreover, to achieve faster bit conversion with an efficient time sequence, asynchronous SAR logic with an internally generated clock is implemented, which avoids the requirement of a high-frequency external clock, as all conversions are carried out in a single clock cycle. The proposed error amplifier-based bandgap reference voltage generator provides a stable reference voltage to the ADC for practical implementation. The measurement results of the proposed SAR ADC, including an on-chip bandgap reference voltage generator, show an Effective Number of Bits (ENOB) of 9.49 bits and Signal-to-Noise and Distortion Ratio (SNDR) of 58.88 dB with 1.2 V of power supply while operating with a sampling rate of 1 MS/s.

## 1. Introduction

For low-power applications, a Successive Approximation Register (SAR) analog-to-digital converter (ADC) is a good choice to obtain successive digital code from an analog input by using the binary search algorithm. Due to its simplicity and power efficiency, SAR ADC is more popular and favorable for comparison with other types of ADCs [1,2,3]. Traditionally, pipeline ADCs have been frequently used for high-speed and medium-resolution data converters. However, the down-scaling of CMOS technologies and reduction in the power supply voltages arouses some significant obstacles for the power-efficient design of pipeline ADCs, because pipeline ADCs have need for high-gain operational amplifiers, which increases the power consumption of pipeline ADCs. In addition to this, it also degrades the swing of amplifiers, which results in the reduction of the Signal-to-Noise Ratio (SNR) for the provided sampling capacitance value. Additionally, the operational amplifier needs to have high DC gain, which lowers the power efficiency because of the low output resistance from short-channel-length devices. On the other hand, SAR ADCs abolish the requirement for an operational amplifier and can attain magnificent power efficiency [4,5]. According to the present trend, SAR ADC provides a sampling speed of several tens of MS/s for medium resolution, which permits the design of low-power and high-performance ADCs. SAR ADCs also have the characteristic of power dissipation being directly related to the sampling rate. These features of SAR ADCs make them the ideal candidate for many applications, such as data signal acquisition, battery management systems, pen digitizers, etc. The simple concept of the SAR ADC is that the analog input will be held by the sample and then be compared with the reference voltage of the ADC, which is an output of the DAC. A stable reference voltage is required for high-resolution ADCs. For SAR ADCs, static bias current is not required in the design of the dynamic comparator [6,7]; hence, the overall power consumption of SAR ADC scales with the sampling rate.

In the SAR ADC, the analog input signal is compared with the reference voltage by a comparator. This reference voltage should be stable and independent of the environmental condition for the stable analog-to-digital conversion [8]. The reference voltage can also be generated by the supply voltage, but it suffers around ±10% of variation [9]; hence, SAR ADC requires a high-precision reference voltage generated by an on-chip bandgap reference voltage generator circuit [10]. The reference voltage cannot vary with the operating conditions, but it can change within the small range of process, voltage, and temperature (PVT) variations [8,11,12]. Furthermore, parasitic inductance affects the reference voltage line and degrades the overall ADC performance when the reference voltage is generated off-chip. In order to achieve the targeted SAR ADC performance, it requires an on-chip reference voltage generator circuit. 

In this work, we present a power-efficient single-ended asynchronous SAR ADC implemented in 130 nm CMOS technology. The proposed dual-path bootstrap switch reduces the sampling nonlinearity. The VCM-based switching sequence is proposed, which reduces the capacitive DAC total capacitance by half due to the additional reference of VCM. For high speed and power efficiency, we implemented a two-stage dynamic latch comparator. To overcome the speed limitation, which is caused by the capacitive DAC settling, an on-chip bandgap reference voltage generator has been implemented. The design details and topology of a dual-path bootstrap switch, capacitive DAC, a two-stage dynamic comparator, asynchronous SAR logic, and an error amplifier-based bandgap reference voltage generator that satisfies the performance requirements are further discussed in detail. 

The proposed single-ended asynchronous SAR ADC architecture is described in Section 2. The sub-blocks of the proposed asynchronous ADC, such as dual-path bootstrap switching, VCM-based capacitive DAC switching, two-stage dynamic latch comparator, asynchronous SAR logic, and error-amplifier-based bandgap reference voltage generator are described in Section 3, and the measurement results are presented in Section 4. Finally, we conclude the paper in Section 5.

## 2. Proposed ADC Architecture

**Figure 1:**
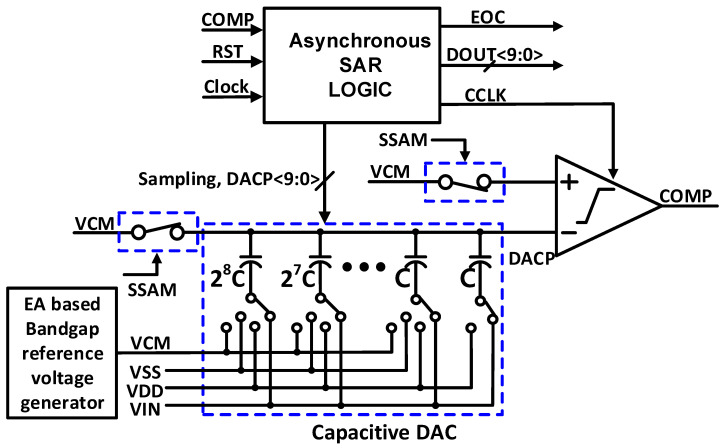
The proposed block diagram of the asynchronous SAR ADC with an EA-based bandgap reference voltage generator circuit.

The proposed single-ended asynchronous SAR ADC topology is designed and fabricated for a 10-bit resolution with a sampling speed of 1 MS/s, and the architecture is presented in Figure 1. The proposed SAR ADC contains a binary weighted capacitive DAC, a bootstrap switch, dynamic comparator, asynchronous SAR logic with an internal comparator clock generator, and a bandgap reference generator. A dual-path bootstrap switch is presented that overcomes the sampling nonlinearity. The VCM-based switching sequence is proposed, which reduces the capacitive DAC’s total capacitance by half due to the additional reference of VCM. Owing not only to the reduced capacitance, but also to the reduced switching step size as well as the removed switching-back operation, the VCM-based CDAC switching achieves excellent energy efficiency. The capacitive DAC is controlled by the digital output code, which is stored by asynchronous SAR logic and the decision made by the comparator. In the proposed single-ended asynchronous SAR ADC, a reference voltage of 0.6 V is generated by the error amplifier (EA)-based bandgap reference voltage generator. The 10-bit capacitive DAC provides the reference voltage DACP to the one input of the comparator, and the other input comparator has common-mode voltage (VCM). The sampling and hold operation is conducted by the sampling switch and capacitive DAC capacitors. The sampling signal, DACP<9:0>, CCLK, and SSAM are the control signals generated from the asynchronous SAR logic. The CCLK signal is provided to the comparator for the fast comparison of the comparator. For high speed and power efficiency, we implemented a two-stage dynamic latch comparator. In addition to this, for practical use, we implemented an on-chip bandgap reference voltage generator that provides better stability and reduced offset voltage distribution.

## 3. Circuit Implementation

### 3.1. Bootstrap Switching

**Figure 2:**
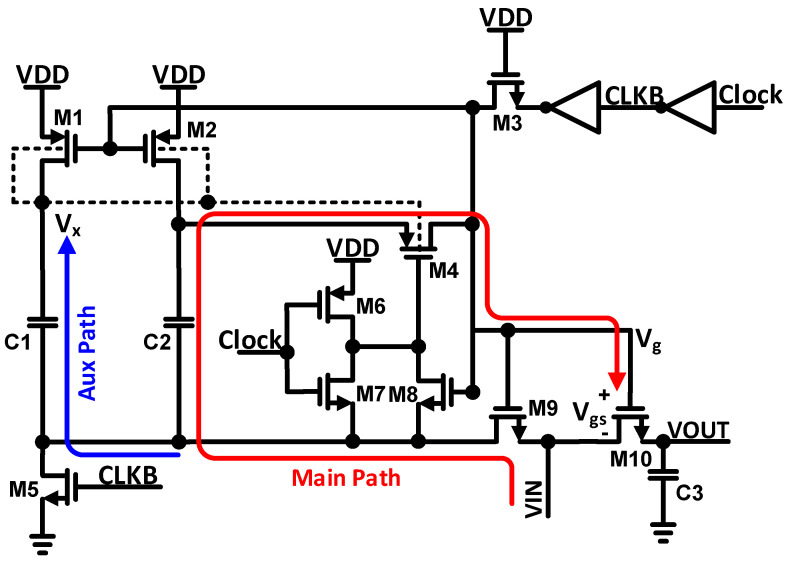
The schematic of the proposed bootstrap switch.

Nowadays, for linear sampling, a bootstrap switch is frequently used, and its non-idealities have become pronounced; thus, its linearity is seriously degrading. Several techniques have been used to improve the performance of the bootstrap switch, such as modifying the circuit network or incorporating fast-turn-on circuits [13]. In Figure 2, we propose a technique to improve the linearity of the bootstrap switch. Ideally, to achieve the constant switch on-conductance, the Vgs of the sampling transistor M10 is independent of and constant with the input. The proposed technique integrates the dual-path bootstrap switch to improve the sampling nonlinearity, and operates at the sampling rate of 1 MS/s, with a 50% duty cycle and peak-to-peak voltage of 600 mV, as shown in Figure 2. This technique creates two paths for the signal; one is the main path, which contains M2 and C2, and the other is an auxiliary path, which contains M1 and C1. In the auxiliary path, the PMOS transistor’s M1, M2, and M4’s bulk terminals are connected to the V_X_ node, which prevents forward biasing. By the proposed dual-path bootstrap switching technique, the nonlinear capacitance drives through the auxiliary path, while the gate of the sampling switch propagates the input signal, and hence the nonlinear capacitance is not directly being loaded into the main path. In this way, we can maximize the drive strength and signal linearity by independently optimizing the auxiliary path and the main path. The formula for voltage transfer to V_g_ from VIN and its phase are expressed as follows:

(1) $$\frac{V_{g}}{\mathrm{VIN}}={\left({\mathrm{sR}}_{4}C_{g}+\frac{C_{g}}{C2}+1\right)}^{-1}$$

(2) $$\phi =\omega_{\mathrm{IN}}R_{4}C_{g}{\left(1+\frac{C_{g}}{C2}\right)}^{-1}$$

where R_4_ = 1/G4, G4 is the on conductance of transistor M4, and Cg is the gate capacitance of transistor M10. By the proposed bootstrap switch, G4 is more linear because, instead of the supply voltage VDD, the bulk of M4 is connected to the nonlinear voltage V_X_. Therefore, the square root of the error of nonlinear voltage V_X_ is directly proportional to G4. Hence, to improve the nonlinearity, the nonlinear voltage V_X_ goes through the bulk of M4, and nonlinear parasitic capacitance from the main path is removed by C2.

### 3.2. Capacitive DAC

Various circuit techniques could further enhance the low power advantage of SAR ADCs. A large amount of research has been conducted to save the CDAC’s switching power consumption. Unlike the traditional SAR ADC architecture with two voltage references, the VCM-based switching scheme proposed could reduce the total capacitance of the CDAC by half due to the additional reference of VCM. It does not only reduce the overall CDAC capacitance, but also reduces the switching step size. With the removed switching-back operation, the VCM-based CDAC switching could achieve excellent energy efficiency and become popular for low-power designs. One drawback of the VCM-based switching scheme might be the difficulty in designing low-resistance switches for VCM. The monotonic switching technique can eliminate the need for VCM by the asymmetric CDAC switching, but this scheme has a varying common-level problem [14,15]. The energy-saving switching technique could implement VCM-based-like switching behavior without utilizing VCM by splitting each capacitor in half. The improved process controllability of advanced CMOS technologies also contributed to reducing the CDAC switching power consumption by decreasing the minimum unit capacitor values without the need for a dedicated process for capacitor implementation.

**Figure 3:**
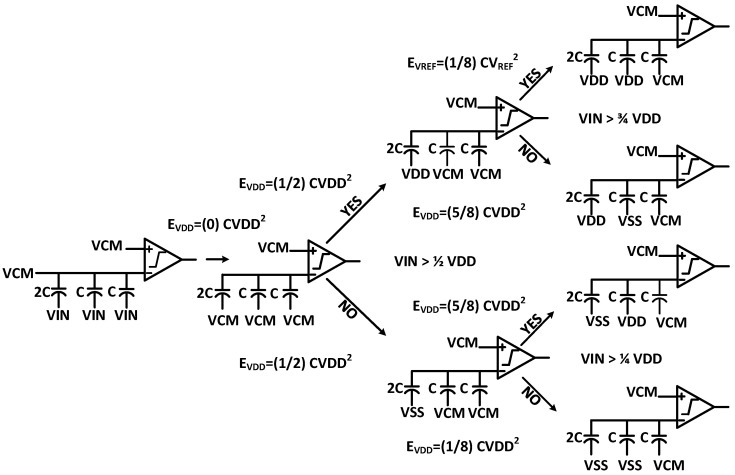
The proposed 3-bit switching method for single-input SAR ADC.

**Figure 4:**
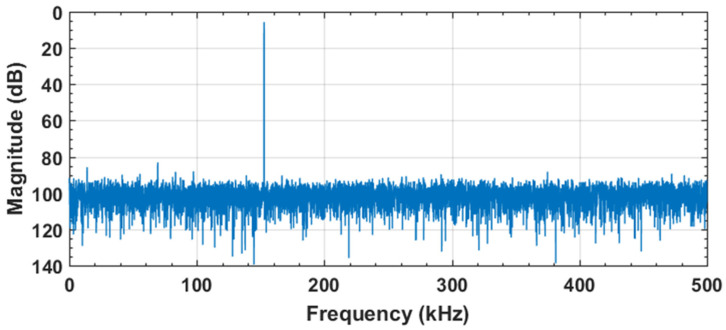
Dynamic performance of proposed switching with 1% unit capacitor mismatch.

**Figure 5:**
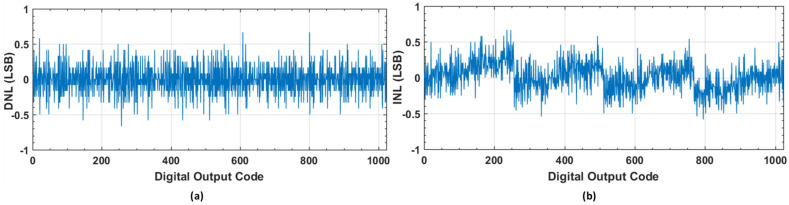
Static performance of the proposed switching with 1% unit capacitor mismatch. (**a**) DNL; (**b**) INL.

The proposed CDAC switching with the binary weighted array used in the single-ended SAR ADC is depicted in Figure 3. In the sampling phase, the bottom plates of all capacitors are connected to the VCM and the top plates are connected to the VIN. Thus, the input voltage is sampled on the binary weighted capacitor array, and we obtain the first signed bit without consuming any switching energy. Depending on the first signed bit, the next conversion cycle is either charged to VDD or discharged to VSS from VCM. Hence, the MSB capacitor is not required in the proposed switching scheme. The sensitivity due to the capacitor mismatch, dynamic, and static performance of the proposed capacitive DAC switching scheme is checked based upon the behavioral simulation in MATLAB. The fast Fourier transform (FFT) spectrum of the behavioral model-level simulation is evaluated in MATLAB^®^ for the proposed switching architecture with 1% unit capacitor mismatch, as shown in Figure 4. The static performance metrics, differential non-linearity (DNL), and the integral non-linearity (INL) of the proposed switching with 1% unit capacitor mismatch are shown in Figure 5a,b respectively.

**Figure 6:**
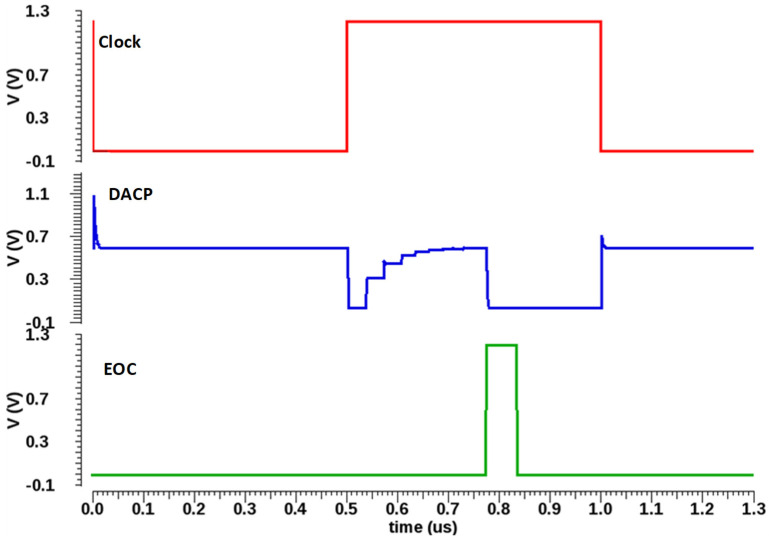
Simulation result of capacitive DAC.

Figure 6 shows the output of the capacitive DAC (DACP) settling of input signals according to the clock signal. An end-of-conversion (EOC) signal will be generated after the completion of the conversion cycle based on the asynchronous SAR logic.

### 3.3. Two-Stage Dynamic Comparator

**Figure 7:**
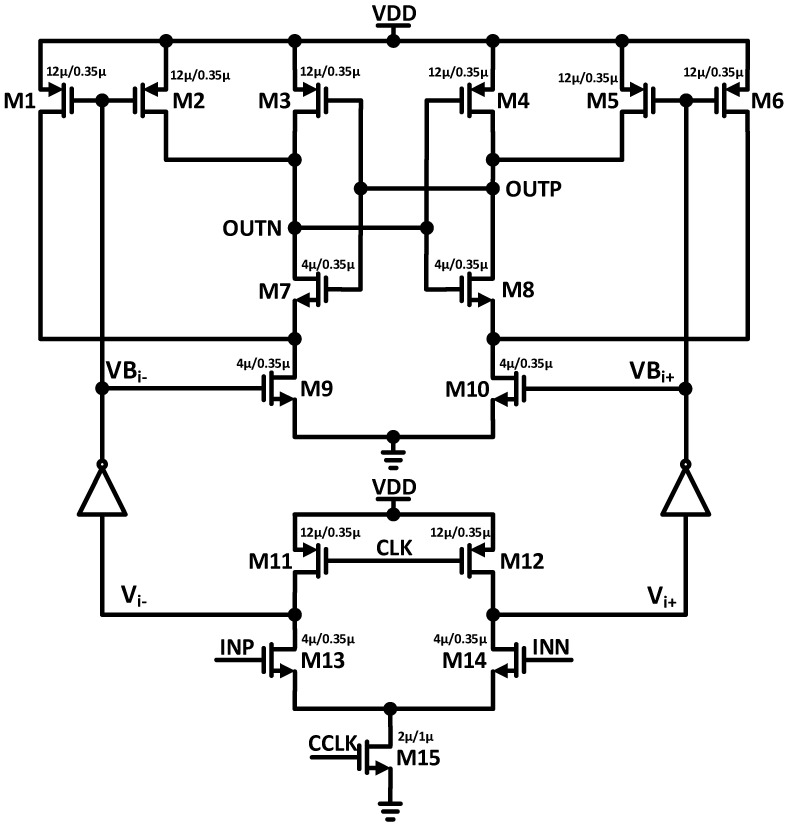
Transistor-level schematic of the dynamic comparator.

In the proposed two-stage dynamic latched comparator, two inverters are added to make the V_i_ node’s voltage strong by providing a higher regeneration speed, as shown in Figure 7. The proposed architecture of the two-stage dynamic latch comparator provides high speed and power efficiency, and lowers the input-referred offset compared with conventional comparator architecture [16,17,18]. When the clock signal CCLK is turned off, the PMOS transistors M11 and M12 are on, then the V_i_ nodes are charged to VDD, and VB_i_ nodes are discharged to VSS during the reset phase. Consequently, there is no static power dissipation and static path due to charge sharing, and no DC flows in the static state of the proposed comparator. The NMOS transistors M9 and M10 drain, and output nodes charge to VDD, while the PMOS transistors of the regeneration stage turn on, and the VB_i_ nodes discharge to VSS.

In the evaluation phase, when the clock signal CCLK increases, the V_i_ nodes discharge to VSS depending on the input voltage through the input transistors M13 and M14, and the tail transistor M15. The VB_i_ nodes are charged from VSS to VDD, while the V_i_ nodes are discharged to VSS in the evaluation phase. Other transistors will be turned on when the NMOS transistors M9 and M10 are turned on in the second stage and either of the VBi nodes reaches the threshold voltage V_th_. Consequently, the latch is activated and regenerates the digital voltage at the output.

### 3.4. Asynchronous SAR Logic and Comparator Clock Generator

To achieve faster bit conversion with an efficient time sequence, asynchronous SAR ADC is more popular [19,20]. Asynchronous SAR logic with an internally generated clock avoids the requirement for the high-frequency external clock, as all conversions are carried out in a single clock cycle. Asynchronous SAR control logic is implemented for a shorter critical path.

**Figure 8:**
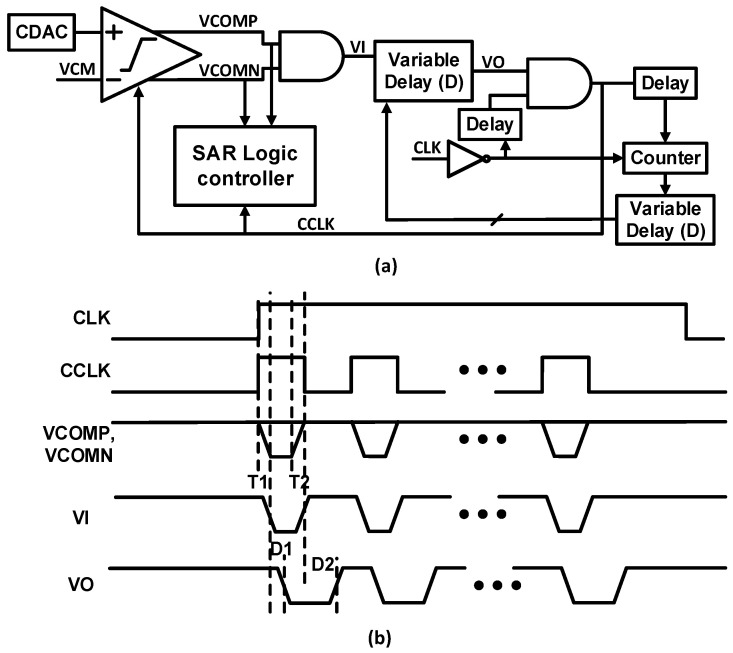
(**a**) Block diagram of the clock generator; (**b**) timing diagram of the clock generator.

A clock generator for SAR control logic is proposed, as shown in Figure 8a. The asynchronous clock generator consists of a delay cell, variable delay, an edge counter, delay adjust block, and logic gates. VCOMP or VCOMN are low, which allows VI to decrease after the decision of the comparator. Then, after the variable delay, VO is also low, which makes the CCLK decrease, and the SAR logic controller is triggered. The comparator starts the comparison when the reset of the comparator is completed, and VI, VO, and CCLK increase. To maximize the sampling period of conversion and to adjust the time delay, the delay adjust block and counter are used in feedback.

**Figure 9:**
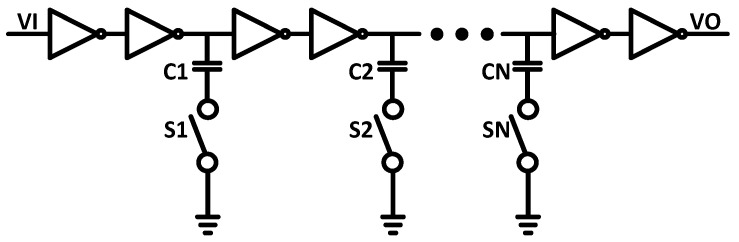
Schematic of variable delay cell.

CCLK provides the reset time of the comparator and more time for DAC settling, as shown in Figure 8b. The proposed clock generator eliminates the memory effect in the comparator and speeds up the bit conversion. Hence, it also helps to improve the ADC robustness. The timing diagram of the proposed clock generator is shown in Figure 8b. The comparator’s decision time and reset time are represented as T1 and T2, respectively. D1 and D2 are the delay time, and unequal D1 and D2 can be obtained by the variable delay cell, as depicted in Figure 9. The variable delay cell is composed of an inverter array, implemented to achieve the desired variable delay. The arrangement of the inverter array leads the delay D1 to be small for the falling edge from VI to VO and delay D2 to be large for the rising edge.

### 3.5. Error Amplifier-Based Bandgap Reference Voltage Generator

**Figure 10:**
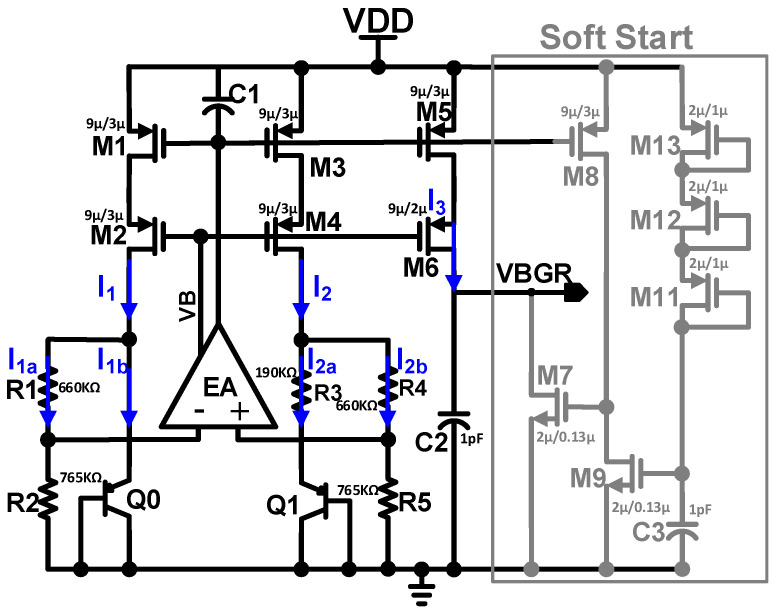
Schematic of BGR.

**Figure 11:**
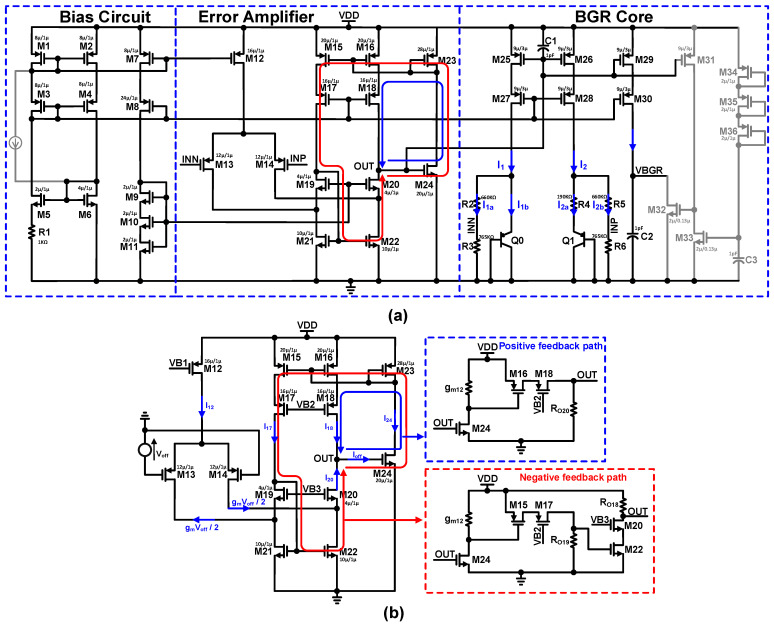
(**a**) Detailed schematic of the proposed error amplifier based bandgap reference voltage generator; (**b**) Proposed folded-Cascoded error amplifier with intentional positive and negative feedback loop.

Reference voltage generators are required to stabilize the overall PVT variation, and also need to be implemented without modifying the fabrication process [21,22,23]. The bandgap reference voltage generator (BGR) is a popular reference voltage generator that successfully achieves the requirements [24,25]. Low power and low voltage operation are the characteristics of reference voltage generators. The error amplifier feedback keeps the same voltage level at both inputs of EA, and R3 generates the voltage difference between the two BJTs, as represented in Figure 10. A Soft-Start circuit is added to the output; when the power signal is high, a current starts to flow through the PMOSs, M11, M12, and M13 connected in diode fashion. To prevent the BGR peak voltage, it slowly charges the capacitor C3, and the BGR output voltage rises. The output of the error amplifier controls the gate of transistors M1 and M3 so that the input voltages of the error amplifier are equal. The positive feedback and negative feedback improve the loop stability of the proposed error amplifier. A detailed schematic of the error amplifier-based BGR circuit is shown in Figure 11a. VB1, VB2, and VB3 are the biasing voltages provided by the bias circuit to the cascaded error amplifier. We assume that the transistors M15–M18 and M23 are matched in terms of their aspect ratios, and the drain current of M18 is represented as:

(3) $$I_{18}=I_{17}=I_{15}=I_{16}=I_{24}=\frac{g_{m24}}{2}\left(\mathrm{OUT}-V_{\mathrm{THn}}\right)=\frac{g_{m16}}{2}\left(V_{ds23}-V_{\mathrm{THp}}\right)$$

Initially, V_off_ caused the output offset current I_off_ of the proposed error amplifier circuit shown in Figure 11b. Between the feedback stage and cascade stage output, OUT triggers the two opposite currents. The offset currents I_18_ and I_20_ are the positive feedback path and negative feedback path, respectively, and act contrary to each other. Therefore, the output offset current I_off_ is expressed as follows:

(4) $$I_{\mathrm{off}}=I_{18}-I_{20}=I_{18}-\left(I_{22}-I_{14}\right)$$

(5) $$I_{\mathrm{off}}=\frac{g_{m16}V_{ov16}}{2}+\frac{g_{m14}V_{\mathrm{off}}}{2}-\frac{g_{m22}V_{ov22}}{2}$$

where V_ov_ is the overdrive voltage; V_ov_ = OUT − V_TH_ and I_20_ = g_m_V_off_/2. By carefully setting the overdrive voltage V_ov_ and transconductance g_m_, we can alleviate the output offset current I_off_, as estimated by Equation (5). We assume that all transistors’ transconductance is the same; then Equation (5) can be expressed as:

(6) $$I_{\mathrm{off}}=\frac{g_{m}}{2}\left(V_{ov16}+V_{\mathrm{off}}-V_{ov22}\right)$$

**Figure 12:**
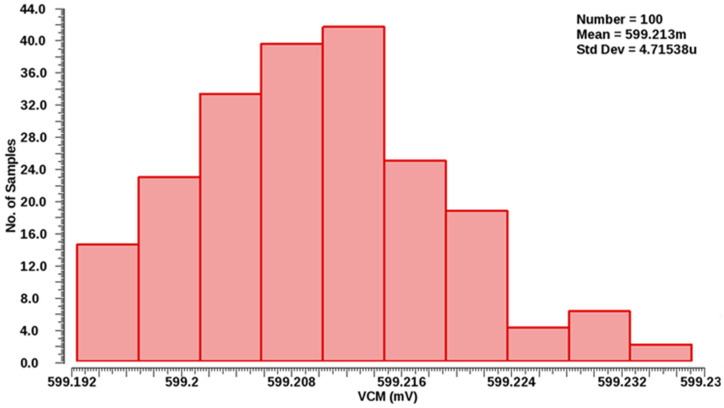
Monte Carlo simulation result of error amplifier-based bandgap reference voltage generator.

Equation (6) represents that we can reduce the output offset current I_off_ by adjusting the V_ov22_ to V_ov16_ + V_off_. This type of reduction in the output offset current I_off_ can be achieved by inducing the intentional feedback loop in the proposed error amplifier. The Monte Carlo simulation of the proposed error amplifier-based bandgap reference voltage generator is depicted in Figure 12. An on-chip voltage generator with a standard deviation of less than 1 LSB is used in the proposed ADC architecture.

## 4. Measurement Results

**Figure 13:**
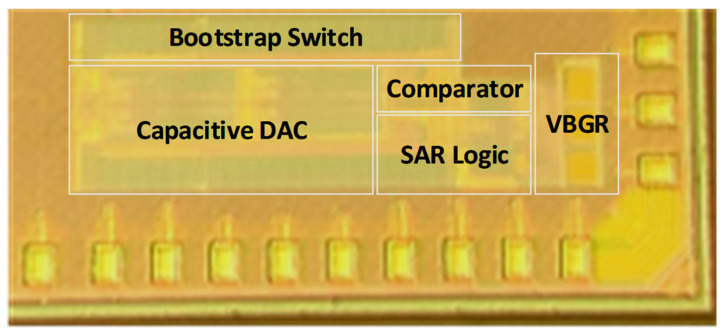
Die photograph of the asynchronous SAR ADC with VBGR.

**Figure 14:**
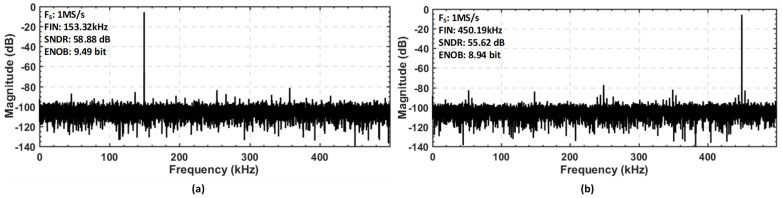
Measured dynamic performance at a sampling speed of 1 MS/s with the two different input frequencies: (**a**) 153.32 MHz input frequency; (**b**) 450.19 MHz input frequency.

The proposed SAR ADC architecture with VBGR was implemented and tested with TSMC 130 nm CMOS technology. Figure 13 represents a die photograph of the asynchronous SAR ADC with VBGR. The measured dynamic performance of the ADC at 153.32 kHz and 450.19 kHz input frequencies with a sampling rate of 1 MS/s is presented in Figure 14a,b, respectively. The proposed SAR ADC architecture achieved 9.49-bit ENOB and 58.88 dB SNDR with an input frequency of 153.32 kHz, as shown in Figure 14a, and 8.94-bit ENOB and SNDR of 55.62 db with an input frequency of 450.19 kHz at 1 MS/s sampling speed, as shown in Figure 14b.

**Figure 15:**
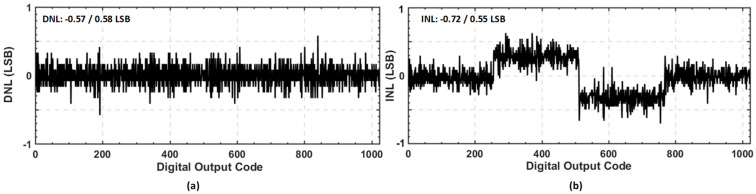
Measured static performance: (**a**) Differential non-linearity (DNL); (**b**) Integral non-linearity (INL).

**Figure 16:**
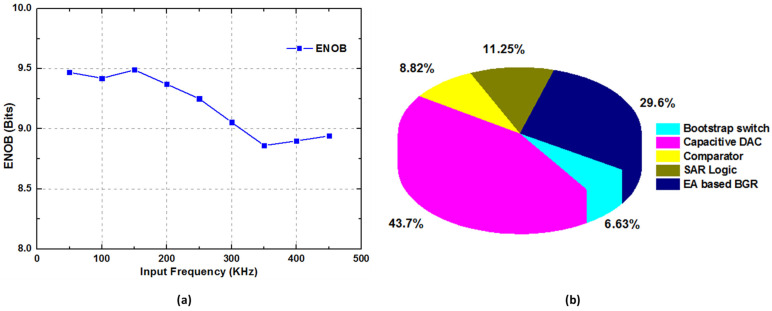
(**a**) Measured ENOB trend at different input frequencies with 1 MS/s sampling speed; (**b**) Power breakdown of proposed ADC.

To check the linearity or the static performance of the proposed SAR ADC, the measured DNL and INL results are presented in Figure 15. The measured DNL and INL were −0.57/0.58 LSB and −0.72/0.55 LSB, respectively. The ENOB trend of the proposed ADC with an on-chip EA-based bandgap reference voltage generator is depicted in Figure 16a,b, representing the power breakdown of the proposed EA-based bandgap reference voltage generator. Table 1 summarizes the performance of the proposed SAR ADC architecture and compares it with the other state-of-the-art SAR ADC architectures. The figure of Merit (FOM) is generally used to check the overall performance of ADC, and the FOM can be evaluated as below:

(7) $$\mathrm{FOM}=\frac{{\mathrm{Power}}_{\mathrm{ADC}}}{\min\left(F_{S},2\times \mathrm{BW}\right)2^{\mathrm{ENOB}}}$$

where the sampling rate is presented as F_S_, bandwidth of ADC is denoted as BW, and power consumed by the proposed ADC is represented as Power_ADC_. The proposed SAR ADC architecture achieved a FOM of 66.25 fJ/conv-step.

## 5. Conclusions

A proposed prototype of a 10-bit 1 MS/s single-ended asynchronous SAR ADC with an on-chip bandgap reference voltage generator is fabricated with 130 nm technology. To optimize the power consumption, static, and dynamic performance several, techniques have been proposed. A dual-path bootstrap switch is proposed to increase the linearity sampling. The VCM-based CDAC switching technique has been implemented for the CDAC part to alleviate the switching energy problem of the capacitive DAC. The proposed architecture of the two-stage dynamic latch comparator provides high speed and low power consumption. Moreover, to achieve the faster bit conversion with an efficient time sequence, asynchronous SAR logic with an internally generated clock is implemented, which avoids the requirement for a high-frequency external clock, as all conversions are carried out in a single clock cycle. The proposed error amplifier-based bandgap reference voltage generator provides stable reference voltages to the ADC for practical implementation. The measurement results of the proposed SAR ADC including an on-chip bandgap reference voltage generator showed an ENOB of 9.49 bits and SNDR of 58.88 dB with 1.2 V of power supply and operation with a sampling rate of 1 MS/s.

## Figures and Tables

**Table 1:** Performance summary and comparison table.

Parameter	[16]	[17]	[14]	[10]	[15]	This Work
Technology (nm)	65	180	180	55	180	130
Resolution (bits)	13	10	8	10	10	10
Supply Voltage (V)	1.2	1.2	1.8	1	1	1.2
Sampling Rate (MS/s)	10	1	1	8	10	1
ENOB (bits)	10.35	8.70	7.23	9.56	9.83	9.49
SNDR (dB)	64.1	54.13	45.3	59.3	60.94	58.88
DNL (LSB)	-	0.4	0.66	−0.2/0.4	−0.3/0.2	−0.57/0.58
INL (LSB)	-	0.46	0.61	−0.6/0.5	−0.3/0.2	−0.72/0.55
Power Consumption (µW)	980	34.6	10.3	572	98	47.64
FOM (fJ/con-step)	71	83	67	94.7	63	66.25

## Data Availability

Not applicable.
